# Vitamin A concentration in bovine liver and milk does not only depend on characteristics of the farming system

**DOI:** 10.1038/s41538-025-00397-9

**Published:** 2025-03-17

**Authors:** Kirsten Schulz, Martin Bachmann, Jens Raila, Ruth Schmitt, Rudolf Staufenbiel, Heiko Scholz, Monika Wensch-Dorendorf, Sebastian Ptok, Anke Weissenborn, Robert Pieper

**Affiliations:** 1 German Federal Institute for Risk Assessment (BfR), Berlin, Germany; 2 Institute for Nutritional Science, University of Potsdam, Nuthetal, Germany; 3 Ruminant and Swine Clinic, Freie Universität Berlin, Berlin, Germany; 4 Professor Hellriegel Institute e.V., Anhalt University of Applied Sciences, Bernburg, Germany; 5 Institute of Agricultural and Nutritional Sciences, Martin Luther University Halle-Wittenberg, Halle (Saale), Germany

**Keywords:** Risk factors, Physiology

## Abstract

The study examined the vitamin A status in commercially managed suckler cows and lactating dairy cows and identified primary influencing factors. Liver retinyl ester concentrations were higher in multiparous than primiparous cows (*p* < 0.01). Pasture availability was associated with higher β-carotene concentrations (*p* < 0.001). In dairy cows, pasture access during the dry period did not affect any of the parameters assayed. β-Carotene and retinol in milk increased with parity. No vitamin A deficiency or hypervitaminosis A was detected. Liver and milk retinol and retinyl ester concentrations that were analysed in the present study and data from a recent German total diet study were used to estimate the exposure to preformed vitamin A in vulnerable groups (children, 0.5–5 years). 95th percentiles of preformed vitamin A intake do not exceed tolerable upper intake levels in individuals between 1 year and 5 years, but in infants 6 to 12 months of age.

## Introduction

Vitamin A describes a group of fat-soluble compounds with retinol activity, which mainly includes retinol and retinyl esters^1^ present in animal-based foods, as well as provitamin A carotenoids such as β-carotene, α-carotene or β-cryptoxanthin that are precursors of vitamin A and are mainly found in yellow and orange fruits and vegetables, but also in feed plants, predominantly in unpreserved forage^2,3^. Feed plants also contain non-provitamin A carotenoids such as lutein and zeaxanthin^4^. The biological value of substances with vitamin A activity is expressed as retinol equivalent (RE), i.e. 1 µg RE is equivalent to 1 µg retinol, 6 µg β‐carotene or 12 µg other provitamin A carotenoids^5^. Alternative to RE, retinol activity equivalents (RAEs) are also used, e.g. by the German Nutrition Society (DGE), to equate different sources to retinol, where 1 µg RAE is equivalent to 1 µg retinol, 12 µg β-carotene or 24 µg other provitamin A carotenoids^6^. Once ingested and released from the feed, lutein and zeaxanthin are specifically transported and accumulate in the macula lutea, whereas provitamin A carotenoids are taken up into the enterocytes of the small intestines and cleaved by β-carotene-15,15’-monooxygenase to retinal. Retinal is then reduced to retinol, from which retinyl esters and retinoic acid are formed^7^. The retinyl esters are packed into chylomicrons together with fat and cholesterol, which enter the lymphatic system and the bloodstream^7^. Lipoprotein lipase hydrolyses the retinyl esters in the chylomicrons and this facilitates uptake by peripheral tissues such as the liver^7^. The most active and abundant form of vitamin A stored in mammalian tissues (and thus also in foods for human consumption) is all-*trans*-retinol, which is particularly present in the esterified forms^8^. In cattle livers and milk, most part (up to 75%) of these esters are retinyl palmitate, followed by retinyl stearate and retinyl oleate^9,10^. Other retinyl esters are not abundant in liver or milk and retinol reaches only 9% of total vitamin A^9,10^. The concentration of vitamin A precursors is negligibly low in tissues and body fluids^11^. The biological functions of vitamin A, i.e. its active forms and metabolites such as retinol, retinoic acid, 9-*cis*-retinoic acid and 13-*cis*-retinol, either in humans or animals, appeal to cellular growth and differentiation, skeletal and foetal development, formation of the central nervous system, corneal and conjunctiva development and the immune response modulation^12–14^. In cattle, and dairy cows specifically, in time clearance of reactive oxygen species that appear at high metabolic rates is of utmost importance to protect animal health and maintain product quality^15^. Vitamin A is discussed to stimulate the defence against oxidative stress^15,16^. That way, it promotes mucosal integrity and stability, e.g. of the mammary gland, to resist pathogen entry and post-entry spread^15,17^. Moreover, vitamin A supports the synthesis of immunoglobulin transport glycoproteins^15,18^. In dairy cows, the Society of Nutrition Physiology (GfE) recommends 5000 IU vitamin A/kg dry matter (DM) during lactation and rearing and twice as much outside lactation^19^. Dairy cows require 40,000 IU of vitamin A per day for maintenance and the gross requirement is 2000 IU/kg milk^19^. In suckler cows, specific recommendations do not exist. Persistently deficient vitamin A supply can have detrimental effects on health status and growth, and could be immunosuppressive^15,20,21^. Parker et al.^22^ reported ataxia, agitation and seizures in vitamin A-deficient calves. Vitamin A overdose instead is unlikely without extreme over-supplementation, because ruminants have a very high tolerance^23,24^. However, excess vitamin A may diminish plasma α-tocopherol concentrations^25^, which is a potent antioxidant^26,27^, and can be associated with premature epiphyseal plate closure in calves that induces abnormal bone growth^28^.

Liver and milk vitamin A levels in cattle seem to be primarily affected by the diet and the feeding system and secondarily by factors related to the animal such as production level, number of days in milk and age^11,29^. Block and Farmer^29^ showed that higher percentages of haylage in the ration increased plasma β-carotene and vitamin A concentrations. Fedele et al.^30^ and Agabriel et al.^31^ have shown that milk retinol concentration increased when cows grazed on pasture.

With regard to human nutrition, most people do not regularly consume liver or liver products^32,33^. However, their proportion in the diet can be high in some areas of the world^34^. Milk consumption is by contrast considerably high in many countries—e.g. in 2023, 23.7 million metric tons in the European Union, 20.9 million metric tons in the U.S.^35^. In humans, vitamin A deficiency is indicated by impaired vision, weight loss, epithelial keratosis and other symptoms summarised by Carazo et al.^13^. Hypervitaminosis A includes toxic and teratogen effects, as well as detrimental effects on bone metabolism^13,36^. The DGE as part of the D-A-CH societies recommends a daily intake of 350 µg RAE for preschool children (4 to less than 7 years), 450 µg RAE for children from 7 to less than 10 years, 600 µg RAE for children from 10 to less than 13 years, 700 µg to 950 µg RAE for adolescents up to 18 years and 700 µg to 850 µg RAE for adults^6^. In pregnancy, it is recommended that women ingest 800 µg RAE per day; during the time of breastfeeding, 1300 µg RAE per day^6^. In Germany, people regularly exceed the recommended levels for vitamin A intake through normal food consumption, and vitamin A deficiency is very uncommon in the German population^32^. The European Food Safety Authority (EFSA) derived a tolerable upper intake level (UL) for the daily intake of preformed vitamin A (i.e. retinol and retinyl esters) of 3000 µg RE for adults, including pregnant women, and of 800–2600 µg RE for younger age groups^5^.

There is a considerable lack of data on the vitamin A status of suckler and dairy cows in modern commercial farms and little is known as to how the vitamin A status is affected by animal and management factors. The objective of the present study was to describe the vitamin A status from a practical survey in commercially managed suckler cow and dairy cow herds and to identify primary influencing factors. Vitamin A deficiency or excess can have a significant health impact, both in the animal and in humans through the consumption of animal products such as liver and milk. Data on vitamin A status collected in the field is, therefore, an important contribution to risk assessment and consumer health protection.

## Results and discussion

### Carotenoids, retinol and retinyl esters in feed

The total mixed rations (TMR) fed to the dairy cows were exemplarily sampled and analysed for carotenoids, retinol and retinyl esters. Lutein, zeaxanthin and β-carotene were found in amounts of 21 ± 6.6, 9.9 ± 4.0 and 11 ± 4.4 µg/g in lyophilized samples. Carotenoids can primarily be found in fresh forages, while concentrates such as cereal grains are low in carotenoid concentration^3^. Prache et al.^37^ reported total carotenoid concentrations of pasture in a range of 430–700 µg/g DM, dependent on vegetation maturity. Pickworth et al.^38^ analysed the provitamin A carotenoids α-carotene, β-carotene and β-cryptoxanthin in feed samples. Fresh fescue pasture had the highest concentration of carotenoids, i.e. β-carotene (100 ± 6.62 µg/g DM), followed by maize silage (17 ± 2.6 µg/g DM), fescue hay (7.3 ± 14 µg/g DM) and wheat straw (0.15 µg/g DM)^38^. Lindqvist et al.^39^ reported β-carotene concentrations in a range of 30–37 µg/g DM in legume-grass silages. In hay and silages, carotenoid concentrations decrease over time from harvest to sample collection^38^. The measured concentrations of β-carotene correspond more to a maize-based than grass-based TMR. In the case of TMR with approximately 80% grass silage (suckler cows), the measured concentrations are therefore rather low. Retinol or retinyl esters have not been detected in the TMR samples, as they are found naturally only in foods of animal origin. Retinol and retinyl esters are thus usually not present in feed, but are formed after ingestion and absorption of the precursors (carotenoids)^7,9^.

### Liver accumulation

**Fig. 1:**
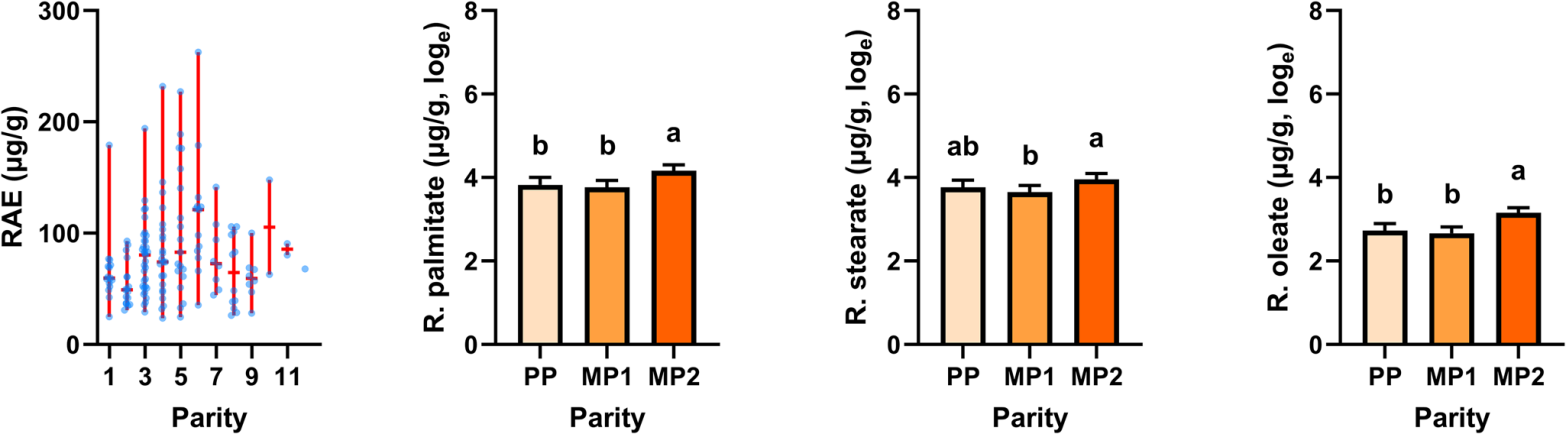
Distribution of RAE calculated on the basis of measured β-carotene, retinol and retinyl ester concentrations and least squares means and standard errors of the concentration of retinyl esters in liver tissue biopsies of suckler cows in relation to parity. In the first chart, the median, min and max are marked by red lines. In charts 2–4, least squares means and standard errors were estimated and are displayed on the basis of data converted to the natural logarithm. RAE retinol activity equivalent, PP primiparous cows, MP1 multiparous cows (lactation no. 2), MP2 multiparous cows (lactation no. 3 and higher). ^a,b^Indicate differences among parities with *p* < 0.05.

**Fig. 2:**
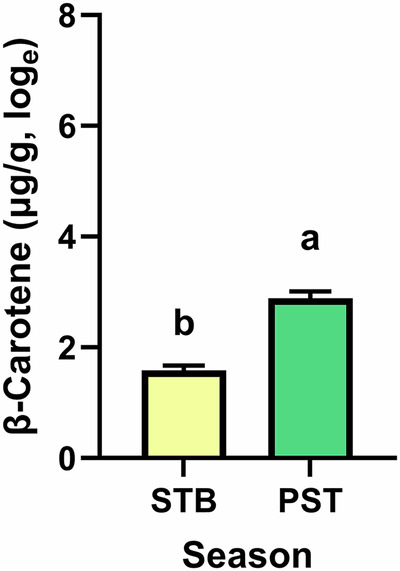
Least squares means and standard errors of the concentration of β-carotene in liver tissue biopsies of suckler cows in relation to the season. The least squares means were estimated and displayed on the basis of data converted to the natural logarithm. STB stable, PST pasture. ^a,b^Indicate the difference between the seasons with *p* < 0.001.

In suckler cows, the concentration of the carotenoids lutein and zeaxanthin in liver tissue were at a maximum of 6.3 µg/g and 0.99 µg/g, respectively, and in most cases not detectable. Liver concentration of β-carotene ranged from 1.7 µg/g to 21 µg/g, corroborating the results of other studies^40^. Stored vitamin A, either expressed as RE or RAE, ranged from 24 to 264 µg/g. Published data comprise a comparable range, i.e. 11–67 µg/g^9^, but individual measures can be much higher^41,42^. As reported by others, retinyl palmitate and retinyl stearate were the most abundant retinyl esters stored in the liver^9,11^. Parity did not affect the liver concentration of β-carotene and retinol (Table 1). However, the concentration of retinyl esters was higher in the liver of multiparous cows in lactation no. 3 than in the liver of primiparous cows (*p* < 0.01; Table 1 and Fig. 1). Such an effect of parity has also been described by Majchrzak et al.^9^. A general decline in vitamin A concentration, at least in blood plasma, occurs in the transition period^43,44^ and this is the sensitive phase in which nutritional intervention is often required. In the summer season, when suckler cows were allowed to graze on pasture, they stored significantly more β-carotene in the liver (*p* < 0.001; Table 1 and Fig. 2). However, we did not detect any seasonal difference in retinol or retinyl ester concentrations, which indicates that the liver pools are not that sensitive to feeding or management effects. Also, Landes^45^ stated that the effects of ration type on vitamin A storage in the liver are not unambiguous. Application of nitrogen fertilizers to the pasture or vitamin A supplementation did not have any effect on liver retinol or retinyl ester concentration (data not shown).

**Table 1:** Descriptive statistics on the concentration of β-carotene, retinol, retinyl esters and vitamin A (RE and RAE) in liver tissue biopsies of suckler cows and the effects of parity and season

	Statistics						Effects (*p* value; *F* test)	
	*n*	Mean	Median	SD	Min	Max	Parity	Season
β-Carotene	153	6.8	6.1	3.3	1.7	21	0.16	<0.001
Retinol	148	1.9	1.8	0.92	0.47	5.0	0.80	0.08
Retinyl palmitate	151	68	61	38	10	218	<0.001	0.30
Retinyl stearate	152	52	48	29	13	187	<0.01	0.53
Retinyl oleate	152	24	20	15	5.8	84	<0.001	0.36
RE	153	81 (269)	72 (239)	44 (144)	24 (79)	264 (881)	<0.001	0.62
RAE	153	80 (267)	71 (237)	44 (144)	24 (79)	263 (875)	<0.001	0.56

Analytes are given as µg/g, RE and RAE additionally as IU/g liver tissue (in brackets).

*RE* retinol equivalent, *RAE* retinol activity equivalent, *SD* standard deviation.

**Fig. 3:**
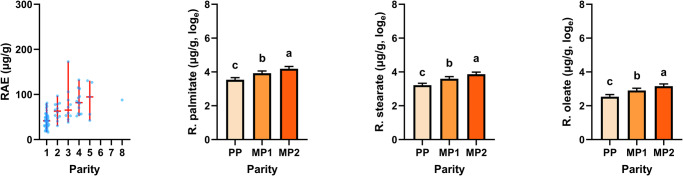
Distribution of RAE calculated on the basis of measured β-carotene, retinol and retinyl ester concentrations and least squares means and standard errors of the concentration of retinyl esters in liver tissue biopsies of dairy cows in relation to parity. In the first chart, the median, min and max are marked by red lines. In charts 2–4, least squares means and standard errors were estimated and are displayed on the basis of data converted to the natural logarithm. RAE retinol activity equivalent, PP primiparous cows, MP1 multiparous cows (lactation no. 2), MP2 multiparous cows (lactation no. 3 and higher). ^a,b,c^Indicate differences among parities with *p* < 0.05.

In dairy cows, the concentration of lutein and zeaxanthin in liver tissue was maximal 3.6 µg/g and 0.50 µg/g, respectively. These analytes were not detectable in most of the samples. The concentration of β-carotene was 0.88–6.7 µg/g, which is in the range given by other studies^40^. Liver vitamin A concentrations ranged from 16 µg/g to 173 µg/g, which is also comparable to published data^9^. Also, in the liver of dairy cows, retinyl palmitate and retinyl stearate were the most abundant retinyl esters. Parity did tendentially affect liver concentrations of β-carotene (*p* = 0.06) and retinol (*p* = 0.07). Liver concentrations of retinyl esters were higher in multiparous than in primiparous cows (*p* < 0.001; Table 2 and Fig. 3). However, the husbandry system—i.e. free-stall housing without or with pasture access to dry cows—did not affect any of the analytes. Although intake of carotenoids should have been elevated during the dry period^37^, this did not have an effect on the liver storage throughout the following lactation period.

**Table 2:** Descriptive statistics on the concentration of β-carotene, retinol, retinyl esters and vitamin A (RE and RAE) in liver tissue biopsies of dairy cows and the effects of parity and the husbandry system

	Statistics						Effects (*p* value; *F* test)		
	*n*	Mean	Median	SD	Min	Max	Parity	Husbandry	Interaction
β-Carotene	78	2.9	2.7	1.2	0.88	6.7	0.06	0.31	0.74
Retinol	77	1.6	1.6	0.90	0.18	4.2	0.07	0.44	0.17
Retinyl palmitate	79	53	47	28	14	145	<0.001	0.74	0.75
Retinyl stearate	79	38	33	20	10	118	<0.001	0.73	0.60
Retinyl oleate	79	19	16	9.7	4.5	57	<0.001	0.67	0.50
RE	79	61 (202)	54 (178)	30 (99.9)	16 (54)	173 (578)	<0.001	0.73	0.80
RAE	79	60 (201)	53 (178)	30 (99.6)	16 (54)	173 (577)	<0.001	0.74	0.79

Analytes are given as µg/g, RE and RAE additionally as IU/g liver tissue (in brackets).

*RE* retinol equivalent, *RAE* retinol activity equivalent, *SD* standard deviation.

### Excretion via milk

Retinol concentrations in milk were mostly lower than 0.01 µg/mL. Similarly low were the concentrations of lutein and zeaxanthin with maximum values of 0.053 µg/mL and 0.006 µg/mL, respectively. The retinyl ester concentration, however, ranged from 0.03 µg/mL to 1.2 µg/mL. Vitamin A concentrations were comparable to those published by Rocchi et al.^10^ or Jin et al.^15^. The concentrations of β-carotene and retinol in milk were higher in cows of third or higher parity than in those that had only been in milk once or twice (*p* < 0.05; Table 3). Retinyl palmitate and retinyl oleate concentrations were not affected by parity. Milk production is increasing until cows pass approximately the fourth lactation^46^. Increasing milk production is accompanied by an increase in DM intake and thus also an increase in the intake of carotenoids^47^. We have not found any effects of pasture allowance on the cows in the dry period (Table 3). However, it has been reported that β-carotene and retinol concentrations can be elevated for a certain period of time in cows fed forage-based diets or with access to pasture^11,31^.

**Table 3:** Descriptive statistics on the concentration of β-carotene, retinol, retinyl esters and vitamin A (RE and RAE) in milk samples of dairy cows and the effects of parity and the husbandry system

	Statistics						Effects (*p* value; *F* test)		
	*n*	Mean	Median	SD	Min	Max	Parity	Husbandry	Interaction
β-Carotene	75	0.13	0.093	0.098	0.020	0.44	0.02	0.82	0.25
Retinol	78	0.006	0.004	0.006	0.001	0.023	<0.001	0.73	0.60
Retinyl palmitate	79	0.31	0.30	0.19	0.017	0.82	0.68	0.89	0.23
Retinyl stearate	78	0.011	0.009	0.0081	0.001	0.033	0.02	0.50	0.95
Retinyl oleate	79	0.11	0.094	0.075	0.011	0.33	0.59	0.95	0.18
RE	79	0.26 (0.87)	0.24 (0.80)	0.17 (0.54)	0.020 (0.066)	0.79 (2.6)	0.57	0.92	0.25
RAE	79	0.25 (0.83)	0.23 (0.77)	0.16 (0.52)	0.020 (0.060)	0.74 (2.5)	0.65	0.93	0.22

Analytes are given as µg/mL, RE and RAE additionally as IU/mL milk (in brackets).

*RE* retinol equivalent, *RAE* retinol activity equivalent, *SD* standard deviation.

### Implication for vitamin A supply of cows

In suckler cows, specific recommendations for the daily vitamin A supply do not exist. Dairy cows, however, should receive 2000 IU vitamin A/kg milk^19^. The median milk yield of the sampled dairy herds was 32 kg per cow and day and this corresponds to a requirement of 64,000 IU vitamin A. Assuming 9 kg liver weight (1.5% of body weight), we found a median of 1.6 million IU vitamin A to be stored in the livers. In the suckler cows, this was 2.1 million IU. The GfE^19^ stated that 2.7 million IU of vitamin A are stored in the liver provided the cows are fed in line with their demands. It can be assumed that nearly all of the stored vitamin A is available to the organism^48^ and additional vitamin A is more or less continuously produced from the ingested precursors. Thus, there was no vitamin A deficiency to expect. A maximum of 5.2 and 7.9 million IU vitamin A was detected in the livers of the dairy and suckler cows, respectively, under the assumption of a liver weight of 9 kg. This is two to three times more than the 2.7 million IU the GfE^19^ considered normal. Due to the high tolerance ruminants naturally have towards vitamin A oversupply, hypervitaminosis is unlikely to occur, even in these cows^23,24^.

### Food and consumer safety

Preformed vitamin A concentrations in milk were found in a range of 1.62–66.8 µg RE/100 g with a mean concentration of 22.9 µg RE/100 g, which is slightly below the mean concentration in milk determined in the BfR MEAL Study (27.8 µg/100 g)^42^, the first German total diet study (TDS). Individuals of the KiESEL study (Childrens’ Nutrition Survey to Record Food Consumption of children from 0.5 to 5 years of age) showed a median total intake of preformed vitamin A of 241 µg RE/day, assuming that the children drink milk with mean concentrations of vitamin A as measured in the present study as part of their normal diet and that all other relevant foods contain levels of preformed vitamin A as detected in the BfR MEAL Study, but that no beef liver is consumed. Older infants aged 6–12 months (*n* = 57) are estimated to have the highest median and 95th percentile (P95) intakes of preformed vitamin A with 337 µg RE/day and 779 µg RE/day, respectively. Across all age groups of the KiESEL study, foods included in the food category ‘milk and milk products’ contributed 34% and thus the most to the intake of preformed vitamin A. The median dietary intake of vitamin A in older infants, young children of 1–3 years and children of 4–6 years utilizes the UL to 56%, 28%, and 23%, respectively. However, the upper tail of the exposure distribution (P95) in the age group of older infants exceeds the UL by 30%.

Preformed vitamin A in beef liver was found in a range of 1602–26,083 µg RE/100 g considering both the suckler cow and dairy cow data sets. The mean and P95 levels in the livers were 7296 µg RE/100 g and 14,558 µg RE/100 g, respectively, which are below the mean concentration of preformed vitamin A in beef liver found in the BfR MEAL Study (15,625 µg/100 g)^42^. Differences between the concentrations of preformed vitamin A in the liver samples from this study and those analysed in the MEAL Study might partly be explained by differences in sample collection and preparation procedures. In this study, liver biopsies from living animals were analysed instead of processed foods as in the MEAL Study. An additional consideration of individual beef liver consumption, detected in the KiESEL study by means of a food propensity questionnaire, combined with the mean levels of preformed vitamin A in beef liver of the present study resulted in an overall median dietary intake of 243 µg RE/day for all individuals of the KiESEL study^33,49^. Using the P95 instead of the mean levels of preformed vitamin A from the present study did not affect the median vitamin A intake of the individuals, because only very few children had eaten beef liver. In addition, the estimated P95 of the preformed vitamin A intake in infants (6–12 months of age), children of age 1–3 years and of age 4–5 years were also nearly unaffected by considering either the mean or the P95 preformed vitamin A liver concentrations taken from the present study. Nonetheless, considering the mean or P95 levels in the liver increased the number of individuals with an intake of preformed vitamin A above the UL from 30 to 31 individuals.

In conclusion, the estimated overall median dietary intake of preformed vitamin A in individuals aged 0.5–5 years did not exceed age-specific ULs when the concentrations of preformed vitamin A in liver and milk from the present study were taken into account together with concentrations of preformed vitamin A measured in all other relevant foods in the recent German TDS. For the considered population groups, the rare consumption of liver, recorded by the food propensity questionnaire for rarely eaten foods, showed only marginal effects on the number of individuals exceeding the UL. Nevertheless, since animal liver is a food with very high levels of preformed vitamin A, it can lead to high vitamin A intake and risk of exceedance of the UL in those who consume liver.

## Methods

### Metadata

The farms that participated in this study were located in Mecklenburg–Western Pomerania (*n* = 2), Brandenburg (*n* = 8), Saxony-Anhalt (*n* = 4), Thuringia (*n* = 1) and Saxony (*n* = 1). The study was conducted after approval by the Saxony-Anhalt Federal Administration Authority (42502-3-877 HSA).

The suckler herds consisted of several breeds, including Angus, Charolais, Simmental and Uckermark cattle. Breed effects could not be addressed on the basis of the current data set and sire information was not available. The parity of the cows ranged from lactation 1–12. In the stable, the cows were fed either a TMR consisting of grass silage (80%) and straw or occasionally maize silage, or exclusively grass silage or hay *ad libitum*. The TMR contained 503 ± 156 g DM/kg, 80 ± 24 g crude ash (CA), 107 ± 22.0 g crude protein (CP), 300 ± 47.0 g crude fibre (CF) and 5.0 ± 0.61 MJ net energy lactation (NEL)/kg DM; given as mean of the respective farms and standard deviation. During the pasture season, additional feeds were omitted. The pastures contained 299 ± 136 g DM/kg, 100 ± 22.0 g CA, 148 ± 50.0 g CP, 278 ± 45.0 g CF and 5.7 ± 0.71 MJ NEL/kg DM; given as the mean of the respective farms and standard deviation. The cows had free access to tap water. In three out of eight farms, pasture management was extensive. These farms either practised organic farming or at least avoided the use of artificial nitrogen fertilizers. Four farms had semi-intensive pasture management. Nitrogen fertilizers were applied to a maximum of 60 kg N/ha. One farm applied more than that. In six out of eight suckler herds, vitamin A was supplemented via licking stones or mineral lick mass, both in the stable and on pasture. Among these, vitamin A was supplemented to 150,000 IU/kg in two herds, to 250,000 IU/kg in three herds and to 800,000 IU/kg in one herd.

Apart from the suckler cow herds, eight commercial dairy farms were monitored. Only lactating dairy cows of the Holstein breed were considered eligible for the study. Milk yields ranged from 25 kg to 34 kg per cow and day on a herd average. Parity ranged from lactation 1–8. The dairy cows were kept in a free-stall housing system with cubicles throughout the year. In four farms, the cows were kept solely in the stable. In the other four farms, dry cows were turned out to pasture for a period of three to six weeks. All cows were fed a TMR based on grass silage, maize silage, different concentrates and mineral mixes, and they had free access to tap water. Basic nutrient concentrations of the TMR were as follows: 405 ± 32.4 g DM/kg, 73 ± 10 g CA, 143 ± 13.0 g CP, 30 ± 3.4 g acid ether extract, 180 ± 22.8 g CF, 258 ± 41.3 g starch, 39 ± 12 g total sugar, 10.9 ± 0.473 MJ metabolizable energy and 6.6 ± 0.34 MJ NEL/kg DM; given as mean of the respective farms and standard deviation.

### Sampling

To account for variable feeding conditions in extensive breeding systems, suckler cow herds were visited twice, first between Sept and Dec 2020, representing the end of the pasture season, and a second time between Jan and Mar 2021, representing the end of the winter feeding period, right before cows were brought back to pasture. During the pasture season, pasture spot samples were cut once a month by hand at 5–6 cm height and subsequently pooled from multiple sampling sites, following a diagonal distribution across the entire area. During the winter season, fresh TMR samples were collected monthly by combining six individual (handful) samples from different parts of the feeding table into one representative bulk sample. Other feeds provided to suckler cows during winter (i.e. components that were fed separately) were sampled accordingly. A total of *n* = 130 pasture samples, *n* = 25 suckler cow TMR samples and *n* = 15 single feed component samples were assessed in this trial.

Considering permanent TMR feeding, indoor housing and, hence, less variability in the diet, dairy herds were only visited once between Dec 2020 and Mar 2021. On the sampling day, freshly mixed lactating-cow TMR was sampled on each farm as described above for suckling herds. A total of *n* = 8 dairy cow TMR bulk samples were analysed in this study. For liver and milk sampling, lactating dairy cows within 100–120 days in milk were enrolled in the trial.

Liver tissue biopsies were generally taken from ten cows out of each herd. Considering missing samples, this actually resulted in *n* = 234 liver biopsies available for analysis (*n* = 154 liver biopsies from suckler cows, i.e. *n* = 78 cows after pasture season, *n* = 76 cows after the winter-feeding period, and *n* = 80 liver biopsies from dairy cows).

Milk samples (*n* = 80) were obtained from the same dairy cows that were used for liver tissue sampling.

Liver biopsies were performed via transcutaneous needle punction similar to Swecker^50^. The surgical site was located a few centimetres dorsal from the intersection of a horizontal line through the greater trochanter of the femur and the eleventh intercostal space, approximately one small hand-width ventral to the margin of the lumbar vertebrae. A 10 × 10 cm square field was dry shaved and subsequently disinfected using 60% alcohol and a 10% iodine solution. Afterwards, 10 mL of a 2% concentrated local anaesthetic (Procaine hydrochloride, i.e. 20 mg Procamidor/mL; Richter Pharma, Wels, Austria) were injected in the intercostal space and infiltrated subcutaneously at the puncture site. After 5 min, a scalpel was used to make a stab incision through the skin at the caudal margin of the eleventh intercostal space. The biopsy trocar with locked stylet was inserted through the incision, aimed towards the left elbow, and then pushed through the intercostal muscle, the peritoneum and the liver capsule until the liver parenchyma was reached, as indicated by a typical “snowball crunching” sound and feel. The stylet was removed from the trocar and the latter was pushed forward to collect liver tissue in its lumen. As soon as a sufficient amount of tissue had been collected, the trocar was retracted from the cow. The incision site was protected with two hubs of iodine solution and left open to heal. Approximately 150 mg of fresh liver tissue per cow was immediately placed on ice for transportation to the laboratory, where samples were stored at −18 °C until analysis.

On the sampling day on dairy farms, during routine morning milking, quarter milk samples were obtained from all cows previously assigned to be part of the trial. After the main milking, from each teat, two to three full streams of rest milk were milked into a sterile sampling tube. In the laboratory, quarter milk samples from each cow were gently mixed and subsequently pooled by pipetting equal volumes (2 mL) of each quarter milk sample into an empty tube in order to create one composite milk sample (8 mL) per cow.

### Chemical analyses

Samples of the TMR and all other feeds were thawed, dried and ground using standard procedures.

Analysis of DM, crude nutrients, starch and total sugar concentration in feeds was performed by a commercial laboratory accredited to DIN EN ISO/IEC 17025:2018 and followed official methods of the Association of German Agricultural Inspection and Research Institutes (methods no. 31.2 and 31.3, respectively, i.e. near infra-red spectroscopy)^51^. Metabolizable energy and NEL were calculated according to the GfE^52^ on the basis of crude nutrient analyses.

Carotenoids and vitamin A (retinol and retinyl esters) in feed, liver and milk samples were determined after organic extraction using a modified gradient reverse-phase high-performance liquid chromatography (HPLC) system (Waters GmbH, Eschborn, Germany). The extraction procedure was adapted to feed, liver and milk samples as follows: 1.5 g lyophilized and finely chopped feed sample was soaked with 2 mL distilled water for 4 h in a polypropylene centrifuge tube at 4 °C. Then, feed samples were homogenized in 5 mL of an extraction mixture of *n*-hexane and isopropanol (3:2 *v*/*v*; stabilized with 0.05% butylhydroxytoluol, BHT) and shaken vigorously for 15 min. The upper organic layer was removed and the aqueous phase was extracted two more times with 5 mL *n*-hexane (0.05% BHT). The supernatants were combined and phase separation was achieved after 30 min by adding 5 mL of 0.1 molar NaCl solution. An aliquot of the combined *n*-hexane layer was dried under nitrogen (30 °C), resuspended in 200 µL of isopropanol and injected into the HPLC system. The extraction procedure of the liver samples was as for the feed samples, but without soaking in distilled water. A quantity of 1 mL of milk sample was extracted with 2 mL of a mixture of *n*-hexane and isopropanol (3:2 *v*/*v*; stabilized with 0.05% BHT) on a shaker for 15 min. After centrifugation (1500 × *g*, 10 min), the upper organic layer was removed and the remaining aqueous phase was extracted again with 2 mL *n*-hexane (0.05% BHT) and the procedure was repeated once more. Then, the organic layers were combined, evaporated under dried nitrogen (30 °C), resuspended in 200 µL isopropanol and injected into the HPLC system. Separation of carotenoids, retinol and retinyl esters was performed on a C30 column (5 µm, 250 by 3.0 mm; YMC, Inc., Wilmington, NC, USA). The solvent system consisted of a 60-min linear gradient from solvent A with methanol/water (90:10, *v*/*v*, 0.4 g/L ammonium acetate) to solvent B with methanol/methyl-tert-butyl-ether/water (8:90:2, *v*/*v*/*v*, with 0.2 g/L ammonium acetate) as the eluent at a flow rate of 0.2 mL/min. The column temperature was set to 20 °C. β-Carotene, retinol and retinyl esters were identified by comparison of retention time with an external standard using a photodiode array detector (Model 996; Waters GmbH, Eschborn, Germany). The quantification was done by measuring the absorption at 450 nm for carotenoids and 325 nm for retinol and retinyl esters. The accuracy and precision of the analyses were verified using standard reference material (SMR 968b, fat-soluble vitamins in human serum; National Institute of Standards and Technology, Gaithersburg, MD, USA). The limit of detection, which was defined as an analyte amount that produces a signal with a S/N ratio of 3, was 0.004 µg/mL for β-carotene and 0.001 µg/mL for retinol and retinyl esters in all sample matrices. The coefficient of variation was lower than 4%.

### Calculations

Retinol equivalents and RAE were calculated as follows: RE (µg/g) = retinol + retinyl oleate/1.92 + retinyl palmitate/1.83 + retinyl stearate/1.93 + β-carotene/6; RAE (µg/g) = retinol + retinyl oleate/1.92 + retinyl palmitate/1.83 + retinyl stearate/1.93 + β-carotene/12; all concentrations considered as µg/g^24^. Vitamin A (IU/g) = RE/0.3 or vitamin A (IU/g) = RAE/0.3. The calculation of preformed vitamin A only includes retinol, retinyl oleate, retinyl palmitate and retinyl stearate with the respective conversion factors, but excludes β-carotene.

### Dietary intakes

Within the KiESEL study^33,49^, food consumption data were collected by food records for infants, toddlers and children from Germany aged 0.5–5 years. Data from 952 individuals were used for estimating the dietary intake of preformed vitamin A. Since food records showed no beef liver consumption as such, data on the frequency of liver consumption (for pig and beef liver as a whole) from a complementary food propensity questionnaire for rarely eaten foods were taken into account^49^. The documented frequencies of liver consumption for 66 individuals (corresponding to 93% non-consumers) were combined with age-specific standard portion sizes of 30 g for infants/toddlers and 35 g for older children, corresponding to recommendations for meat portions in childcare facility catering^53^. Data on upper-bound preformed vitamin A levels in foods were taken from the first German TDS (BfR MEAL Study)^42^, whereby the dietary intakes of preformed vitamin A were calculated for a predominant selection of conventionally produced foods^42^. The levels of preformed vitamin A in beef liver and bovine milk from the TDS were replaced by the mean or P95 levels for liver and mean levels for milk obtained from the present study. For the individuals of the KiESEL study, the long-term daily food consumption of each TDS food was multiplied by the corresponding level of preformed vitamin A taken from the TDS data set or the present study as described above. The partial intakes were summed up for each individual. These calculations were done using the R software package (version 4.1.3).

### Statistical analysis

Statistical analysis was performed using SAS 9.4 (SAS Institute Inc., Cary, NC, USA). In the data sets, outliers were identified using boxplots and removed. Outliers were defined as observations more than three times the interquartile range. Additionally, missing values were removed. In the suckler cow data set, this led to 18 out of 1386 values (154 samples × 9 analytes), which were removed. In the dairy cow data set, 27 out of 720 values (80 samples × 9 analytes) were removed (12 from liver and 15 from milk analyses). The cows’ parity was grouped as follows: group 1, primiparous cows, group 2, cows in lactation no. 2 and group 3, cows in lactation no. 3 or higher. Least squares means were estimated for the concentration of β-carotene, retinol, individual retinyl esters and vitamin A (RE and RAE) in liver tissue and milk using the MIXED procedure and the following linear models: for suckler cows, *Y*_*ijklmn*_ = *µ* + *α*_*i*_ + *β*_*j*_ + *γ*_*k*_ + *δ*_*l*_ + *F*_*m*_ + *A*_*n*_ + *ε*_*ijklmn*_, in which *µ* is the general mean, *α*_*i*_ is the fixed effect of parity *i* (*i* = 1, …, 3; 1 = group 1, 2 = group 2, 3 = group 3), *β*_*j*_ is the fixed effect of season *j* (*j* = 1, 2; 1 = stable, 2 = pasture), *γ*_*k*_ is the fixed effect of pasture fertilization *k* (*k* = 1, 2; 1 = not applied, 2 = applied), *δ*_*l*_ is the fixed effect of vitamin A supplementation *l* (*l* = 1, 2; 1 = not applied, 2 = applied), *F*_*m*_ is the random effect of farm *m* (*m* = 1, …, 8), *A*_*n*_ is the random effect of animal *n* (*n* = 1, …, 99 in the final data set; *n* = 96 in retinol, *n* = 98 in retinyl oleate and *n* = 99 in the other analytes), and *ε*_*ijklmn*_ is the random residual effect with *ε*_*ijklm*_ ~ N(0,σ^2^*ε*); for dairy cows, *Y*_*ijk*_ = *µ* + *α*_*i*_ + *β*_*j*_ + *αβ*_*ij*_ + *F*_*k*_ + *ε*_*ijk*_, in which *µ* is the general mean, *α*_*i*_ is the fixed effect of parity *i* (*i* = 1, …, 3; 1 = group 1, 2 = group 3, 3 = group 3), *β*_*j*_ is the fixed effect of husbandry system *j* (*j* = 1, 2; 1 = free-stall with cubicles, without pasture, 2 = free-stall with cubicles, with access to pasture for dry cows), *αβ*_*ij*_ is the interaction of fixed effects *α*_*i*_ and *β*_*j*_, *F*_*k*_ is the random effect of farm *k* (*k* = 1, …, 8) and *ε*_*ijk*_ is the random residual effect with *ε*_*ijk*_ ~ N(0,σ^2^*ε*_*i*_) for liver and *ε*_*ijk*_ ~ N(0,σ^2^*ε*_*j*_) for milk samples. The numbers of animals given in the suckler cow model description are explained by the fact that for 54 animals, observations were available for seasons 1 and 2 and for 45 animals, observations were available for either season 1 or 2. Some of the cows were culled due to farm management decisions. Among the animals with two observations, it happened a few times that birth had not yet taken place on the second measurement date and the animals were therefore continued with the same lactation number. In these cases, the lactation number was set to the subsequent lactation. The concentrations of lutein and zeaxanthin in the liver and milk were extraordinarily low or were not detectable, which is why they did not undergo statistical analysis. Differences in least squares means with *p* < 0.05 were considered to be significant. Tukey–Kramer-adjusted *p* values were displayed for the main effects. In order to obtain normality of the distribution of studentized residuals, the data were transformed to the natural logarithm prior to least squares means estimation. The normality of the distribution of studentized residuals was then confirmed using the UNIVARIATE procedure.

## Data Availability

The data sets used and/or analysed during the current study are available from the corresponding author upon reasonable request.
